# Regulation of the Properties of the Hierarchical Porous Structure of Alumophosphate Molecular Sieves AEL by Reaction Gels Prepared with Different Templates

**DOI:** 10.3390/gels11040297

**Published:** 2025-04-17

**Authors:** Arthur R. Zabirov, Dmitry V. Serebrennikov, Rezeda Z. Kuvatova, Nadezhda A. Filippova, Rufina A. Zilberg, Olga S. Travkina, Marat R. Agliullin

**Affiliations:** 1 Institute of Petrochemistry and Catalysis, Ufa Federal Research Centre of the Russian Academy of Sciences (UFRC RAS), 450075 Ufa, Russiafilippova-ink@yandex.ru (N.A.F.);; 2 Department of Analytical Chemistry, Ufa University of Science and Technology, 450076 Ufa, Russia

**Keywords:** molecular sieves, AlPO-n alumophosphate, materials with hierarchical porous structure, adsorption of paraffins

## Abstract

Microporous alumophosphate molecular sieves AlPO_4_-n are promising materials for use in catalysis, gas adsorption, and gas separation. However, AlPO_4_-n faces problems such as diffusion limitations that lead to a deterioration in mass transfer. To solve this problem, we studied the crystallization of alumophosphate reaction gels prepared using aluminum isopropoxide and various secondary amines as templates, including diethyl-, di-n-propyl-, diisopropyl-, and di-n-butylamines. Using X-ray diffraction, Ramon spectroscopy, and STEM methods, it has been demonstrated that the reaction gels prepared using DPA, DIPA, and DBA are amorphous xerogels consisting of 5–10 nm nanoparticles. The reaction gel prepared with DEA is a combination of a layered phase and an amorphous aluminophosphate. It has been shown that the use of aluminum iso-propoxide allows the production of AlPO_4_-11 in the form of 2–4 µm aggregates consisting of primary AlPO_4_-11 nanocrystals. The template was found to exert a significant effect upon both the characteristics of the porous structure and the size of AlPO-11 nanocrystals. A template is proposed for the synthesis of hierarchical AlPO_4_-11 with a maximum volume of mesopores. The morphology and crystal size of AlPO_4_-11 were found to strongly influence its adsorption properties in the adsorption of octane.

## 1. Introduction

Molecular sieves currently play a key role in developing advanced catalysts and adsorbents [1]. Among the diverse family of molecular sieves, alumophosphates (AlPO_4_-n) and their derivatives (MeAlPO_4_-n, SAPO-n) have attracted particular attention due to their unique properties [2,3]. AlPO_4_-n alumophosphates are a class of microporous materials with a structure formed by alternating AlO_4_ and PO_4_ tetrahedral units linked by common oxygen atoms. These materials exhibit a wide range of structural diversity, with pore sizes ranging from 3.8 Å in AlPO_4_-18 to 12.7 Å in AlPO_4_-8, and spatial organization of channels that can be 1D (AlPO_4_-5), 2D (AlPO_4_-40), or 3D (AlPO_4_-46) [4].

Due to these properties, these materials are actively studied and used in various fields. They are used for adsorption [5,6,7,8,9], encapsulation of laser dyes [5,6,10,11,12], separation of gases (such as CO_2_/CH_4_, CH_4_/N_2_, and Kr/Xe) [13,14,15], catalysis [16,17,18], and development of sensors [19]. They are also used in the development of single layer carbon nanotubes [16] and energy storage and heat conversion systems [20,21]. The introduction of transition metals (Rh, Pd, Fe, Co, Mn, Zn, etc.) into the AlPO_4_-n structure allows for the creation of catalytic systems for various chemical processes [3], such as isomerization, oxidation, alkylation [3], hydroxylation [22], conversion of methanol to olefins [23], and dehydration of 2-propanol [24].

Among the AlPO_4_-n aluminophosphate, molecular sieves with the AlPO_4_-11 (AEL) structure are of particular interest. The materials and their Si-containing derivatives (SAPO-11) exhibit high selectivity for higher C_7+_ n-paraffin. This is due to their channel structure, which creates a one-dimensional system and pores up to 0.5 nm [25,26,27,28,29].

One of the main obstacles to improving the performance of adsorbents and catalysts made from materials with the AEL structure is the restricted diffusion within the microporous framework, resulting in a decrease in mass transfer. In an effort to reduce diffusion limitations, methods have been developed to create nanoscale and hierarchical molecular sieves with an AEL-type structure [30,31,32,33,34]. However, the effects of the chemical nature of the templates on the morphology and properties of the resulting crystals are still poorly understood [35,36]. These factors play a crucial role in determining the adsorption and catalytic properties of these materials.

Recent studies have demonstrated the crucial role played by templates in the formation of molecular sieves. They are involved in the formation of crystal lattice and help to stabilize charges [37]. Changing the concentration of the template affects the pH of the reaction medium, which is important for the creation of a specific crystal structure. In particular, it has been found that the successful crystallization of AlPO_4_-11 molecular sieves requires the presence of secondary amines, which provide the desired electronic effect [38]. For example, diethyl-, di-n-propyl-, di-isobutyl-, di-n-butyl-, n-butyl-, and n-hexylamines were investigated for their potential role in the synthesis of AlPO_4_-11. However, only di-n-propylamine and di-isobutylamine met the necessary criteria as their molecules had sizes that matched the interatomic distances in the zeolite unit cell (about 8.4 Å). This allowed the successful preparation of pure AlPO_4_-11 in high yield.

Similar studies have been carried out during the crystallization of SAPO-11 using various templates including diethylamine, di-n-propylamine, di-isopropylamine, and combinations thereof [36,39,40]. It was found that the best results were obtained using di-n-propylamine and a mixture of diethylamine and diisopropylamine. However, the use of pure diisopropylamine or diethylamine resulted in the formation of SAPO-5 impurities or non-porous aluminophosphates. Subsequent research confirmed these findings and emphasized the importance of selecting the appropriate template to obtain high quality SAPO-11 [41,42]. Previously, we have shown that the preparation of SAPO-11 with high phase purity is feasible using different templates, such as dialkylamines, when the reaction gel subjected to pre-aging is used for its synthesis [43]. In addition, the influence of the template on the morphology, crystal size, and properties of the porous structure of SAPO-11 was shown. The templates allowing the synthesis of SAPO-11 in the form of nanocrystals were proposed.

Previously we have shown that it is possible to synthesize AlPO_4_-11 aluminophosphate with a hierarchical porous structure from reaction gels prepared using Al isopropoxide as a source of aluminum [44,45,46,47]. However, despite the extensive data on the effect of templates on the phase purity of AEL-type materials, there are still insufficiently explored questions regarding the effect of templates on the properties of the reaction gels formed and the characteristics of AlPO_4_-11 crystals. These parameters have a significant influence on their adsorption properties, especially in the process of adsorption of higher C_7+_ paraffins. Following up on the work of [43,47], we hypothesize that using different templates to synthesize AlPO_4_-11 aluminophosphate will allow us to control the porous structure properties in hierarchical AlPO_4_-11.

The present research seeks to thoroughly investigate these factors in order to gain a deeper comprehension of the processes that lead to the creation of AlPO_4_-11 and to refine the conditions for its production to enhance the performance of adsorbents derived from it.

## 2. Results and Discussion

In our previous research, we demonstrated the feasibility of synthesizing AlPO_4_-11 molecular sieves with a hierarchically porous structure, thereby eliminating the necessity for crystal growth modifiers and surfactants [48]. To synthesize hierarchical materials, aluminum isopropoxide was used as a source of aluminum during the preparation of the reaction mixture. Aluminum isopropoxide, due to its high reactivity at the stage of preparation of the reaction gel, makes it possible to obtain clusters of nanocrystals that form a hierarchical porous structure. This suggests that the future properties of AlPO_4_-11 are already determined at the reaction gel preparation stage. Consequently, the study of the physicochemical properties of reaction gels obtained using various templates is imperative for understanding their effect on the characteristics of crystallization products.

**Figure 1:**
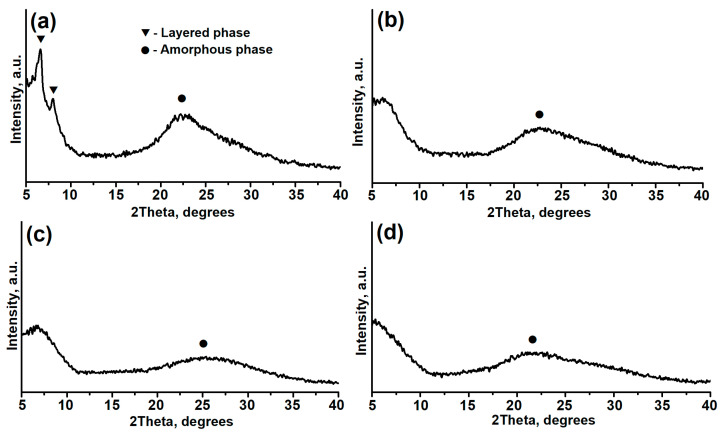
X-ray images of dried reaction gels prepared using aluminum isopropoxide and various secondary amines: (**a**)—AlPO-iAl-DEA; (**b**)—AlPO-iAl-DPA; (**c**)—AlPO-iAl-DIPA; (**d**)—AlPO-iAl-DBA.

Figure 1 shows X-ray images of dried reaction gels obtained using different templates (secondary amines). X-ray analysis shows that gel samples synthesized with di-n-propylamine, di-isopropylamine, and di-n-butylamine all exhibit a wide halo in the region of scattering angles between 20° and 30° 2θ. This is characteristic of amorphous structure formation. However, the X-ray image of the sample obtained using di-ethylamine not only shows the amorphous halo as indicated, but also additional reflections at angles of 6.5° and 8.0°. This indicates the formation of an additional, layered phase. It has been reported in the literature [49] that layered aluminophosphates have a well-ordered structure mainly along the a-b plane, forming thin two-dimensional plates connected by weak van der Waals forces, structurally resembling an AEL-type lattice. These results highlight the template’s substantial impact on reaction gel properties during their synthesis.

**Figure 2:**
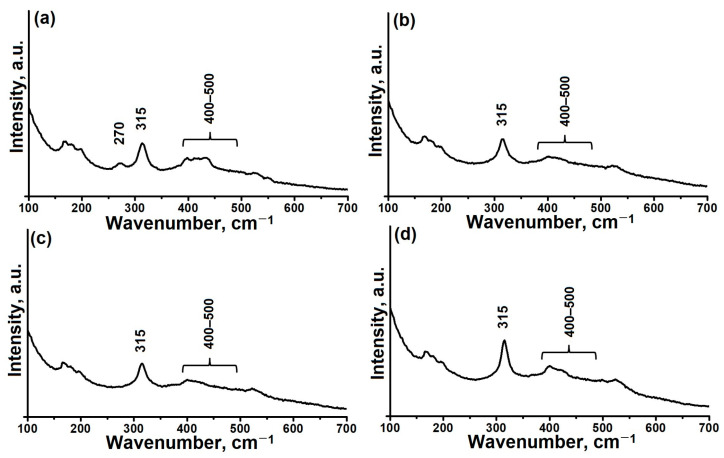
Raman spectra of dried reaction gels prepared using aluminum isopropoxide and various secondary amines: (**a**)—AlPO-iAl-DEA; (**b**)—AlPO-iAl-DPA; (**c**)—AlPO-iAl-DIPA; (**d**)—AlPO-iAl-DBA.

The use of a highly reactive aluminum precursor, such as aluminum isopropoxide, can help to form secondary building blocks (SBU) at the early stages of gel formation [50]. To better understand the properties of the gels that were formed, we collected Raman spectra, as shown in Figure 2. The main peaks observed in these spectra are at 315 and 400–500 cm^−1^, and they are characteristic of amorphous reaction gels. Peaks in the range of 400–500 cm^−1^ are traditionally attributed to 4-R and 6-R structures, while the peak at 315 cm^−1^ corresponds to the tetrahedral (AlO_4_) structure [50,51]. An increase in the molecular weight of the secondary amine, accompanied by a decrease in peak intensity at 400–500 cm^−1^, indicating a reduction in the interactions between aluminum and phosphorus. This could indicate a possible decrease in SBU. In the case of a sample of the AlPO-iAl-DEA reaction gel obtained using diethylamine, an additional peak was detected at 270 cm^−1^, which is usually associated with fluctuations in the 10-R rings [51,52]. The presence of a peak at 270 cm^−1^ in the AlPO-iAl-DEA sample suggests the presence of structural fragments similar to the AEL lattice even in the initial stages of reaction gel synthesis. Comparison of Raman spectroscopy data with X-ray diffraction results suggests that the layered phases have a structure similar to alumophosphate zeolites of the AlPO_4_-11 type. This is because they contain the same structural elements, 4-R, 6-R, and 10-R rings, however the concentration is much lower than in the layered phases.

These observations correlate with an increase in the molecular weight of the hydrocarbon radical in secondary amines, which leads to an increase in their basicity. An increase in molecular weight may increase the interaction between the amine and phosphoric acid, resulting in the formation of a less reactive amine phosphate, which may reduce the likelihood of further reaction with aluminum compounds.

**Figure 3:**
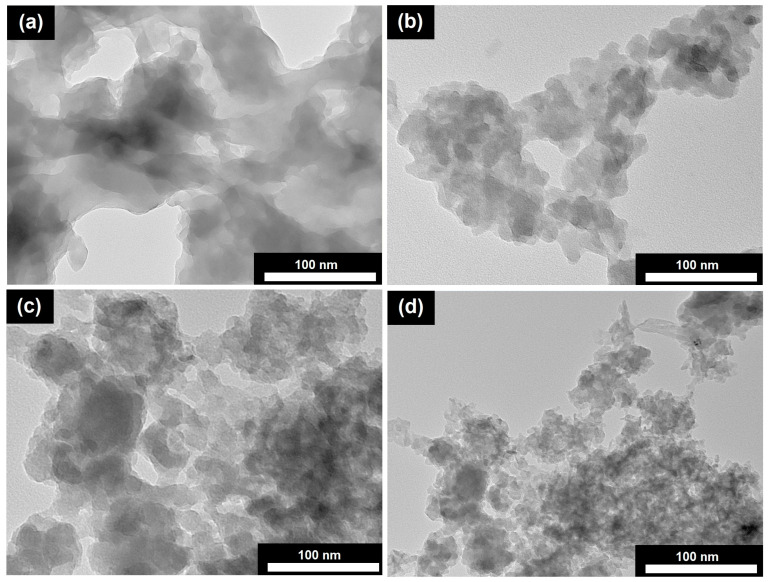
STEM images of dried reaction gels prepared using aluminum isopropoxide and various secondary amines: (**a**)—AlPO-iAl-DEA; (**b**)—AlPO-iAl-DPA; (**c**)—AlPO-iAl-DIPA; (**d**)—AlPO-iAl-DBA.

Figure 3 shows transmission electron microscopy (STEM) micrographs of dried reaction gels. The samples (AlPO-iAl-DPA, AlPO-iAl-DPA, and AlPO-iAl-DBA) show a structure similar to that of xerogels, consisting of aggregates of spherical amorphous particles with diameters between 2 and 10 nm. The AlPO-iAl-DEA reaction gel sample has a different morphology from the others. Its structure consists of a mixture of lamellar structures of about 300 nm in size and spherical particles of 5 to 10 nm in diameter. X-ray diffraction data suggest that the spherical particles are amorphous alumophosphates, while the lamellae may represent layered phases.

Thus, the data obtained from X-ray diffraction analysis, Raman spectroscopy, and transmission electron microscopy indicate that the template used in the preparation of the reaction gel significantly affects its phase composition, the extent of interaction between aluminum and phosphorus sources, and the microstructural characteristics of the formed particles.

**Figure 4:**
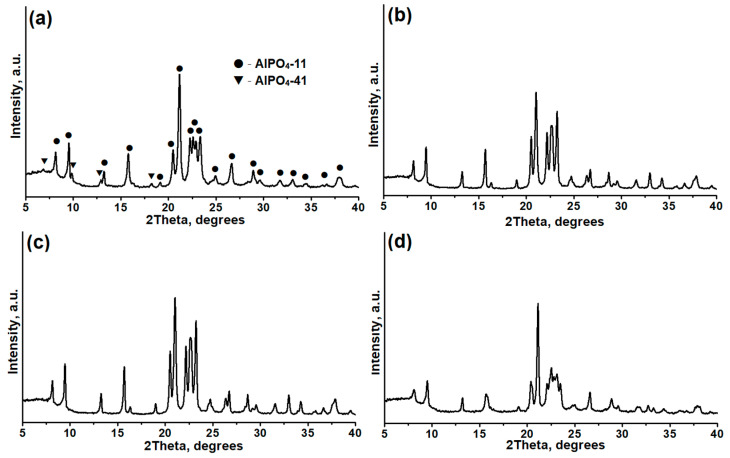
X-ray images of AlPO_4_-11 samples prepared using aluminum isopropoxide and various secondary amines: (**a**)–AlPO-11-DEA; (**b**)–AlPO-11-DPA; (**c**)–AlPO-11-DIPA; (**d**)–AlPO-11-DBA.

Figure 4 shows X-ray images of crystallization products from alumophosphate reaction gels prepared with different templates. Phase composition, crystallinity, and template content in the unit cell are presented in Table 1. Regardless of the template used, the primary product of crystallization for all gels is alumophosphate molecular sieve AlPO_4_-11. In the case of the AlPO4-11-DEA sample, small amounts of AlPO_4_-41 are formed. We have previously shown that DEA, under certain crystallization conditions, allows obtaining AlPO-41 of high phase purity [53]. AlPO_4_-11 samples prepared with di-n-propylamine and diisopropylamine show the highest degree of crystallinity compared to other amines. The findings of this study are in opposition to the results reported in [38], which indicated that the effective synthesis of molecular sieves of AEL type requires the presence of a secondary amine. The length of the amine molecule should be comparable to the distance of the c-axis in the unit cell of the zeolite (about 8.4 Å). Only di-n-propylamine and diisopropylamine meet the criteria. At the same time, our experiments show the possibility of synthesizing AlPO_4_-11 molecular sieves using all the secondary amines examined in this study. It is evident that the phase purity of molecular sieves of the AEL structure is influenced by both the properties of the template and the aluminum source. In most studies, boehmite has been used for the synthesis of these molecular sieves. However, it appears that aluminum isopropoxide may be a more suitable aluminum source, as it allows for the production of AlPO_4_-11 using any secondary amine.

Table 2 summarizes the chemical compositions of the reaction gels and their crystallization products, as determined by XRF analysis. The data reveal that the P/Al ratio in the molecular sieves approaches 1 after crystallization, confirming the high crystallinity of the synthesized samples.

**Figure 5:**
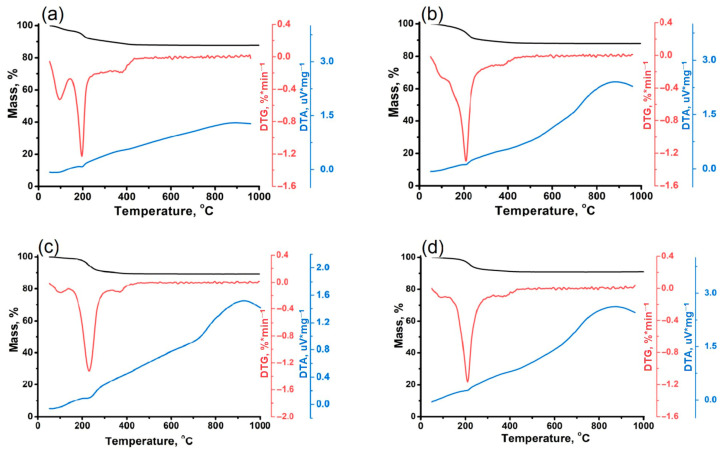
TG/DTG/DTA curves of non-calcined AlPO_4_-11 samples synthesized by various secondary amines: (**a**)—AlPO-11-DEA; (**b**)—AlPO-11-DPA; (**c**)—AlPO-11-DIPA; (**d**)—AlPO-11-DBA.

Thermogravimetric analysis was used to quantify the content of different matrices in AlPO_4_-11 molecular sieves. The heat treatment conditions that ensure complete removal of mines were also determined. The results of the DTG-DTA analysis are shown in Figure 5. Table 3 shows the calculated values for the amount of SDA in a unit cell. Analysis of the TG and DTG curves shows the presence of two distinct stages of mass loss in all the AlPO_4_-11 studied. Intracrystalline water desorption occurs at a temperature of 50–100 °C. The desorption of physically adsorbed secondary amine molecules has been observed to occur within the temperature range of 150–300 °C (Table 3). Endothermic effects are observed on the DTA curves in the temperature range of 150–300 °C (Table 3). These effects indicate the desorption of physically adsorbed molecules. Previously, in [54,55], it was shown that molecular sieves with the AEL structure are thermally stable up to 800 °C and the current thermogravimetric results correspond with this.

With an increase in the molecular weight of the amine, a decrease in the molar ratio of SDA/Unit Cell (structure-forming agent/unit cell) in the AlPO_4_-11 molecular sieve is observed. The maximum value of SDA/Unit Cell was observed for the sample AlPO-11-DEA, and the minimum value was observed for the sample APO-11-DBA.

**Figure 6:**
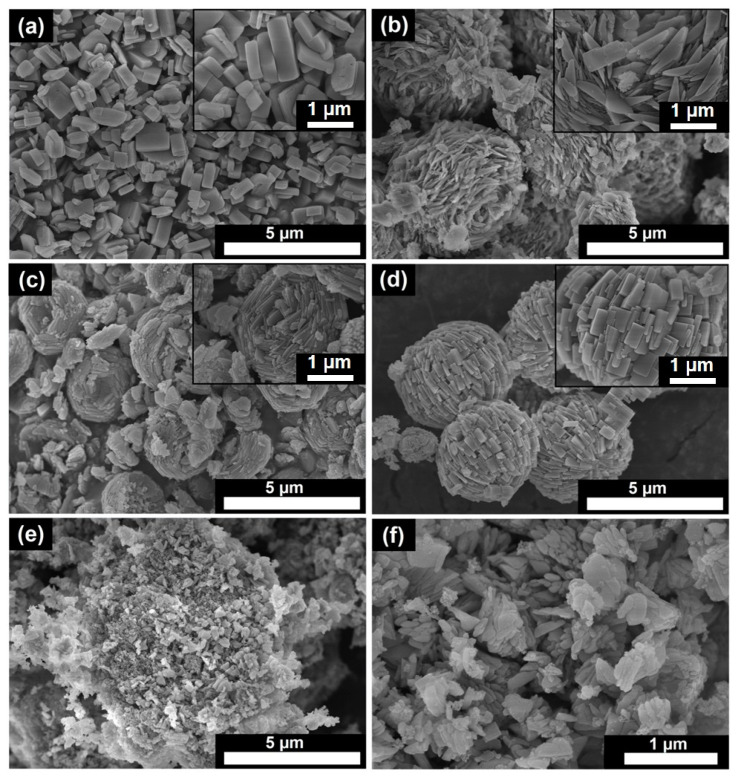
SEM images of AlPO_4_-11 samples synthesized by various secondary amines: (**a**)—AlPO-11-DIPA-micro; (**b**)—AlPO-11-DEA; (**c**)—AlPO-11-DPA; (**d**)—AlPO-11-DIPA; (**e**)—AlPO-11-DBA ×8000; (**f**)—AlPO-11-DBA ×30,000.

The adsorption properties of AlPO_4_-n molecular sieves are strongly determined by their crystal morphology and size [56]. Figure 6 shows scanning electron microscope (SEM) images of samples of AlPO_4_-11 synthesized using different templates and aluminum sources. All the samples synthesized with aluminum isopropoxide appear to be aggregates of nanocrystals.

The samples AlPO-11-DEA, AlPO-11-DPA, and AlPO-11-DIPA are all spherical aggregates with a diameter between 2 and 4 µm. The aggregates of AlPO-11-DEA are formed by flat nanocrystals resembling bowls with a thickness of about 200 nm and a length of about 500 nm. The aggregates in the AlPO-11-DPA sample, on the other hand, are made up of elongated prisms with a thickness of about 100 nm and a length of about 500 nm. Finally, the nanocrystals in the AlPO-11-DIPA sample are in the form of cubic prisms measuring about 300 by 500 nm. The aggregates of the sample, synthesized using di-n-butylamine, are characterized by an irregular shape and a size of approximately 5 µm. These aggregates are formed from flat nanocrystals with a size between 100 and 200 nm. AlPO_4_-11 alumophosphate, also known as AlPO-11-DIPA-micro sample, was synthesized as a reference sample. Bohemite was used as a source of aluminum, and di-isopropylamine served as a template. It can be seen that the synthesized sample does not form splices and consists of rectangular prisms with a size of approximately 1 μm due to the use of boehmite, as confirmed by our previous research [47].

**Figure 7:**
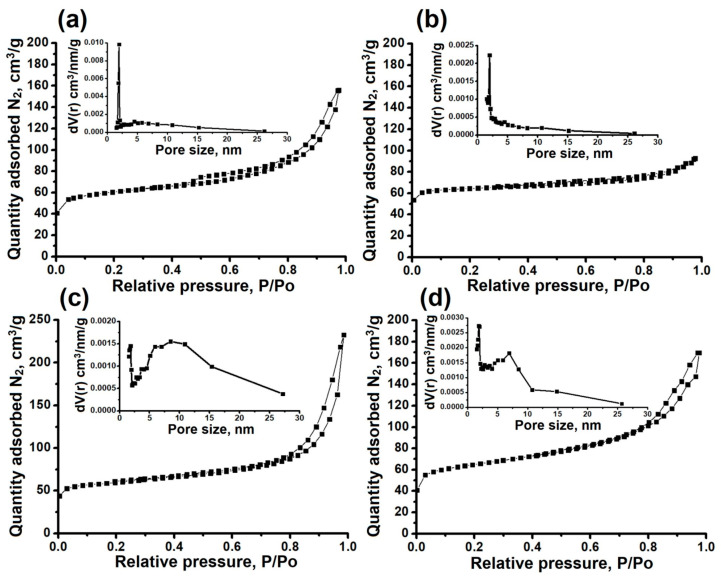
N_2_ adsorption-desorption isotherms and pore size distribution of AlPO_4_-11 samples synthesized by various secondary amines: (**a**)—AlPO-11-DEA; (**b**)—AlPO-11-DIPA; (**c**)—AlPO-11-DPA; (**d**)–AlPO-11-DBA.

The N_2_ adsorption-desorption isotherms and pore size distributions of AlPO_4_-11 molecular sieves prepared with various templates are presented in Figure 7. The textural properties of the synthesized samples are shown in Table 4. All samples prepared with aluminum isopropoxide display a combination of type I and IV-like isotherms featuring H3-type hysteresis loops. This type of isotherm is typical of micro-mesoporous materials. From the pore size distribution, it can be seen that the mesopore diameter is mainly between 2 and 25 nm. Mesopores in these samples are formed due to incomplete fusion of nanoscale crystals, which are observed in SEM images (Figure 6). The AlPO-11-DBA sample has the largest external surface area (138 m^2^/g) due to the formation of its porous structure by the aggregation of smaller nanocrystals. The mesopore volume for this sample is 0.20 cm^3^/g.

The lowest external specific surface area (58 m^2^/g) and mesopore volume (0.06 cm^3^/g) among the samples obtained with isopropoxide are observed in the AlPO-11-DIPA sample. This is associated with the formation of a secondary porosity due to the growth of nanocrystals with the largest sizes. The reference sample (AlPO-11-DIPA-micro) has the even smaller specific surface area and mesopore volume, as it is characterized by the largest crystal size and almost complete absence of crystal clusters. Thus, we see that by using different secondary amines, it is possible to fine-tune the properties of the porous structure by altering both the specific surface area and the volume of the secondary mesopores.

**Figure 8:**
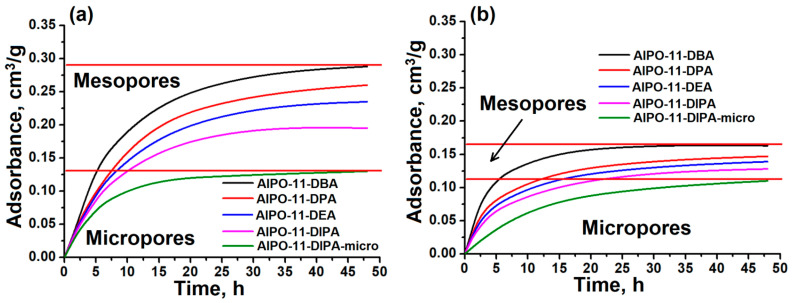
Kinetics of n-octane and iso-octane adsorption on alumophosphate molecular sieves: (**a**) n-octane adsorption; (**b**) iso-octane adsorption.

As mentioned above, AlPO_4_-11 is a promising material for adsorption of higher n-paraffins due to its porous structure (10R-1D). Figure 8 shows the results of adsorption of n-octane and isooctane on synthesized micro-mesoporous molecular sieves and a microporous control sample.

It can be seen that by using different secondary amines, it is possible to fine-tune the properties of the porous structure by changing both the specific surface area and the volume of the secondary mesopores. In all cases, the adsorption rate of n-paraffin molecules is greater than that of isooctane. This can be explained by the larger size of the isooctane molecules, which causes additional diffusion limitations. Samples with a micro-mesoporous (hierarchical) structure show a higher adsorption rate compared to a microporous reference sample (AlPO-11-DIPA-micro).

Complete saturation of the micropores with octane molecules is achieved within 5–10 h for micromesoporous AlPO_4_-11, whereas it takes 10–40 h for isooctane. These differences can be explained by the reduced crystallite sizes in the hierarchical AlPO_4_-11 samples. The developed secondary porosity favors the acceleration of diffusion and adsorption processes. In particular, the AlPO-11-DBA sample has the fastest micropore filling. This is associated with the smallest primary crystal size and the most developed secondary porosity. On the other hand, the AlPO-11-DIPA sample has the lowest micropore filling rate among the hierarchical AlPO_4_-11 samples. This is due to the formation of secondary crystals from larger primary structures in the form of elongated prisms, as well as a lower proportion of transport mesopores in the secondary structure. It should also be noted that the saturation of the pores with n-octane molecules in the microporous 11-DIPA-micro sample occurs only after 40 h, while complete adsorption of iso-octane on this material is not observed even after 48 h. Therefore, the formation of a hierarchically organized porous structure in molecular sieves of the AlPO_4_-11 type plays a crucial role in the development of highly efficient adsorption materials of the new generation.

## 3. Conclusions

In this study, we have investigated the crystallization of alumophosphate reaction gels using aluminum isopropoxide and different templates, such as diethylamine (DEA), di-n-propylamine (DPA), diisopropylamine (DIPA), and di-nbutylamine (DBA), into AlPO_4_-11 molecular sieves with a hierarchical porous structure.

It has been found that the structure and size of the template used has a considerable influence on the phase composition of the reaction gels formed. Gels synthesized using DPA, DIPA, and DBA are amorphous materials with a spherical aggregate size of 2–4 µm.

The spherical aggregates of the AlPO-11-DEA sample consist of flat, bowl-shaped nanocrystals with a thickness of about 200 nm and a length of about 500 nm. The aggregates of the AlPO-11-DPA sample are composed of elongated prisms with a thickness of about 100 nm and a length of about 500 nm, while the aggregates of the AlPO-11-DIPA sample are in the form of cubic prisms measuring 300 by 500 nm. Finally, the aggregates of the AlPO-11-DBA sample have an irregular shape and measure about 4 µm. They are composed of nanocrystals with dimensions of 100 to 200 nm.

It has been shown that increasing the molecular weight of the template reduces the strength of the interaction between the aluminum and phosphorus sources.

Crystallization of gels with a composition of 1.0Al_2_O_3_·1.0P_2_O_5_·1.0SDA·40H_2_O, regardless of the type of template used, has been shown to produce AlPO_4_-11 molecular sieves with high phase purity.

It has been established that the structure of the template influences crystal size, morphology, and the characteristics of the secondary porosity of AlPO_4_-11. The use of DBA as a template allows the formation of AlPO_4_-11 with a highly developed secondary porous structure (S_EX_ = 138 m^2^/g, V_meso_ = 0.20 cm^3^/g). The synthesis of AlPO_4_-11 revealed an inverse correlation between amine molecular weight and SDA/unit cell ratio, with AlPO-11-DEA exhibiting the highest template density per unit cell.

The synthesized AlPO_4_-11, with its hierarchical porous structure, exhibits significantly higher adsorption rates for higher n-paraffins compared to conventional microporous materials.

The results of this study suggest the possibility of controlling the morphology and crystal size in AlPO4-11 molecular sieves by template tuning, opening new avenues for the development of effective adsorbents for a new generation of applications.

## 4. Materials and Methods

### 4.1. Preparation of Aluminophosphate Gels

For the synthesis of AlPO_4_-11 molecular sieves, alumophosphate reaction gels were prepared with the following molar composition, 1.0Al_2_O_3_·1.0P_2_O_5_·1.0SDA·40H_2_O, where SDA is a template. Aluminum isopropoxide (i-Al, 99%, Acros Organics, Noisy-le-Grand, France) was used as the aluminum source. Orthophosphoric acid (H_3_PO_4_, 85%, Reachim, Moscow, Russia) was used as the source of the phosphorus. The following amines were used as templates: diethylamine (DEA, 99%, Sigma-Aldrich, Darmstadt, Germany), di-n-propylamine (DPA, 99%, Acros Organics, Schwerte, Germany), di-isopropylamine (DIPA, 99%, Sigma-Aldrich, Darmstadt, Germany), and di-n-butylamine (DBA, 99%, Sigma-Aldrich, Darmstadt, Germany).

The reaction gels were prepared by adding 10.0 g and 27.0 g of orthophosphoric acid and distilled water, respectively. The calculated amount of each template was then added according to the composition of the reaction mixture. The templates used were DEA (3.2 g), DPA (4.4 g), DIPA (4.4 g), and DBA (5.6 g). In addition, 17.7 g of aluminum isopropoxide was added to the resulting suspension with vigorous stirring. The gels prepared using the DEA, DPA, DIPA, and DBA templates were named AlPO-iAl-DEA, AlPO-iAl-DPA, AlPO-iAl-DIPA, and AlPO-iAl-DBA, respectively.

### 4.2. Crystallization of AlPO_4_-11 Molecular Sieves

Molecular sieves of the AlPO_4_-11 type are prepared by hydrothermal synthesis using reaction gel heated at 200 °C for 24 h. After the hydrothermal synthesis, the AlPO_4_-11 suspension was centrifuged to isolate the solid product from the mother solution. The collected precipitate was washed repeatedly with deionized water to eliminate residual reagents and then dried at 80–100 °C until constant weight was attained. The crystallization yield for each sample was determined by weighing the dried powder and comparing it with the theoretical yield calculated from the reaction stoichiometry. The samples obtained with different additives are designated AlPO-11-DEA, AlPO-11-DPA, AlPO-11-DIPA, and AlPO-11-DBA.

### 4.3. Material Analysis Methods

A Shimadzu XRD 7000 diffractometer (Shimadzu Corporation, Kyoto, Japan), operating in the CuKa radiation range, was used to determine the phase composition of dried gels and their crystallization products. Scanning was performed over a range of 2θ from 5 to 40°, with an increments of 1° per minute. The X-ray images data processing and phase analysis was performed using Shimadzu XRD software in conjunction with the PDF2 database (version 2.2201). The degree of crystallinity was assessed by analyzing the amorphous halo content over the angle range of 2θ between 20° and 30° using the Shimadzu XRD Cristalinity software (version 7.04).

The chemical composition of the reaction gels and aluminophosphate molecular sieves was determined using X-ray fluorescence spectroscopy on a Shimadzu EDX-7000P spectrometer (Shimadzu Corporation, Duisburg, Germany).

An FT-Raman NXR 9650 Fourier spectrometer (Thermo Scientific, Waltham, MA, USA) was used to record the Raman spectra. The spectra of aluminophosphates were recorded in the wave number range between 70 and 800 cm^−1^ with a resolution of 2 cm^−1^.

The morphological characteristics and size of the intermediate phases were analyzed using scanning transmission electron microscopy (STEM) and the crystal structures were investigated using scanning electron microscopy (SEM) on a Regulus SU 8220 (Hitachi, Tokyo, Japan) using the secondary electron registration mode (accelerating voltage is 5 kV).

To determine the content of templates and heat treatment conditions for AlPO_4_-11 molecular sieves for complete removal of amines, the method of thermogravimetric analysis on a synchronous thermal analyzer STA 449 F5 (Netzsch, Selb, Germany) was used. The study was conducted in the temperature range of 50–1000 °C, with programmable heating at a rate of 10 °C/min. The analysis was performed in a corundum crucible in a helium atmosphere, with a sample weight of 20–30 mg.

The micro- and mesopore volumes were calculated using low-temperature N_2_ adsorption-desorption on a Quantachrome Nova 1200e sorbtometer (Quantachrome Instruments, Boynton Beach, FL, USA). The specific surface area was determined using the Brunauer–Emmett–Teller (BET) method. The volume of micropores in the presence of mesopores was estimated by the t-Plot method. The pore size distribution was determined using the Barrett–Joyner–Halenda (BJH) model along the desorption branch after calcining the samples at 600 °C for 5 h prior to measurements.

The adsorption kinetics of hydrocarbons, including n-octane and isooctane, were studied using a Hiden Isochema IGA001 (Hiden Isochema, Warrington, UK) gravimetric gas absorption analyzer. The experiments were carried out at a temperature of 25 °C and an atmospheric pressure of 101,380 Pa.

## Figures and Tables

**Table 1:** Phase composition, degree of crystallinity, and crystallization yield of AlPO_4_-11 molecular sieves prepared using various templates.

Sample	Phase Composition	DC, %	Crystallization Yield, %
AlPO-11-DEA	AEL (90%) + AFO (10%)	-	90
AlPO-11-DPA	AEL	92	89
AlPO-11-DIPA	AEL	91	85
AlPO-11-DBA	AEL	92	86

DC—Degree of crystallinity, %.

**Table 2:** Chemical composition of reaction gels and AlPO_4_-11 molecular sieves.

Sample	Gel	AlPO_4_-11
AlPO-11-DEA	Al_1.00_P_0.99_	Al_1.00_P_0.98_
AlPO-11-DPA	Al_1.00_P_0.97_	Al_1.00_P_0.99_
AlPO-11-DIPA	Al_1.00_P_0.99_	Al_1.00_P_0.99_
AlPO-11-DBA	Al_1.00_P_0.97_	Al_1.00_P_0.98_

**Table 3:** TG/DTG/DTA analysis results and template content in the unit cell of AlPO_4_-11 molecular sieves prepared using various templates.

Sample	M, %	Q, µV/mg	SDA/Unit Cell
AlPO-11-DEA	7.50	0.087	4.1
AlPO-11-DPA	9.22	0.124	3.2
AlPO-11-DIPA	8.44	0.122	3.0
AlPO-11-DBA	7.18	0.275	2.1

M—Quantity of adsorbed secondary amine molecules, %; Q—Endothermic effects on the DTA curves in the temperature range of 150–300 °C.

**Table 4:** The porous structure characteristics of aluminophosphate molecular sieves AlPO_4_-11 synthesized by various secondary amines.

Sample	S_BET_, m^2^/g	S_EX_, m^2^/g	V_micro_, cm^3^/g	V_meso_, cm^3^/g
AlPO-11-DEA	196	113	0.05	0.17
AlPO-11-DIPA	202	58	0.07	0.06
AlPO-11-DPA	225	96	0.07	0.25
AlPO-11-DBA	213	138	0.05	0.20
AlPO-11-DIPA-micro	195	-	0.07	0.05

S_BET_—specific surface according to BET; S_EX_—external specific surface area; V_micro_—specific volume of micropores; V_meso_—specific volume of mesopores.

## Data Availability

The original contributions presented in the study are included in the article. Further inquiries can be directed to the corresponding author.

## References

[1] Vermeiren W., Gilson J.-P. (2009). Impact of Zeolites on the Petroleum and Petrochemical Industry. Top. Catal..

[2] Potter M.E. (2020). Down the Microporous Rabbit Hole of Silicoaluminophosphates: Recent Developments on Synthesis, Characterization, and Catalytic Applications. ACS Catal..

[3] Hartmann M., Elangovan S.P. (2010). Catalysis with Microporous Aluminophosphates and Silicoaluminophosphates Containing Transition Metals. Advances in Nanoporous Materials.

[4] Baerlocher C., McCusker L.B., Olson D.H. (2007). Atlas of Zeolite Framework Types.

[5] Endregard M., Nicholson D.G., Stöcker M., Beagley B. (1995). Cobalticenium Ions Adsorbed on Large-Pore Aluminophosphate VPI-5 Studied by X-Ray Absorption, ^13^C Solid-State NMR and FTIR Spectroscopy. J. Mater. Chem..

[6] Ganschow M., Schulz-Ekloff G., Wark M., Wendschuh-Josties M., Wöhrle D. (2001). Microwave-Assisted Preparation of Uniform Pure and Dye-Loaded AlPO_4_-5 Crystals with Different Morphologies for Use as Microlaser Systems. J. Mater. Chem..

[7] García-Carmona J., Fanovich M.A., Llibre J., Rodríguez-Clemente R., Domingo C. (2002). Processing of Microporous VPI-5 Molecular Sieve by Using Supercritical CO_2_: Stability and Adsorption Properties. Microporous Mesoporous Mater..

[8] Van Heyden H., Mintova S., Bein T. (2006). AlPO-18 Nanocrystals Synthesized under Microwave Irradiation. J. Mater. Chem..

[9] Shutilov R.A., Grenev I.V., Kikhtyanin O.V., Gavrilov V.Y. (2012). Adsorption of Molecular Hydrogen on Aluminophosphate Zeolites at 77 K. Kinet. Catal..

[10] Weiß Ö., Loerke J., Wüstefeld U., Marlow F., Schüth F. (2002). Host–Guest Interactions and Laser Activity in AlPO_4_-5/Laser Dye Composites. J. Solid. State Chem..

[11] Yao M., Wang T., Yao Z., Duan D., Chen S., Liu Z., Liu R., Lu S., Yuan Y., Zou B. (2013). Pressure-Driven Topological Transformations of Iodine Confined in One-Dimensional Channels. J. Phys. Chem. C.

[12] Guo J., Wang C., Xu J., Deng F., Yan W., Sharma R.P., Xu R. (2018). Encapsulation of Bulky Solvent Molecules into the Channels of Aluminophosphate Molecular Sieve and Its Negative Influence on the Thermal Stability of Open-Framework. Inorg. Chem. Commun..

[13] Carreon M.L., Li S., Carreon M.A. (2012). AlPO-18 Membranes for CO_2_/CH_4_ Separation. Chem. Commun..

[14] Wu T., Lucero J., Zong Z., Elsaidi S.K., Thallapally P.K., Carreon M.A. (2018). Microporous Crystalline Membranes for Kr/Xe Separation: Comparison Between AlPO-18, SAPO-34, and ZIF-8. ACS Appl. Nano Mater..

[15] Wang B., Gao F., Zhang F., Xing W., Zhou R. (2019). Highly Permeable and Oriented AlPO-18 Membranes Prepared Using Directly Synthesized Nanosheets for CO_2_ /CH_4_ Separation. J. Mater. Chem. A.

[16] Yang W., Sun W., Zhao S., Yin X. (2016). Single-Walled Carbon Nanotubes Prepared in Small AlPO_4_-5 and CoAlPO-5 Molecular Sieves by Low-Temperature Hydrocracking. Microporous Mesoporous Mater..

[17] Liu D., Zhang B., Liu X., Li J. (2015). Cyclohexane Oxidation over AFI Molecular Sieves: Effects of Cr, Co Incorporation and Crystal Size. Catal. Sci. Technol..

[18] Esther Leena Preethi M., Umasankari A., H.Rekha C., Palanichamy M., Sivakumar T., Pandurangan A. (2018). Selective Oxidation of Cyclohexane to KA Oil Over Ce-Alpo-18 Molecular Sieves. Int. J. Eng. Technol..

[19] Chen Y., Zhang Y., Li D., Gao F., Feng C., Wen S., Ruan S. (2015). Humidity Sensor Based on AlPO_4_-5 Zeolite with High Responsivity and Its Sensing Mechanism. Sens. Actuators B Chem..

[20] Ristić A., Logar N.Z., Henninger S.K., Kaučič V. (2012). The Performance of Small-Pore Microporous Aluminophosphates in Low-Temperature Solar Energy Storage: The Structure–Property Relationship. Adv. Funct. Mater..

[21] Henninger S.K., Jeremias F., Kummer H., Schossig P., Henning H.-M. (2012). Novel Sorption Materials for Solar Heating and Cooling. Energy Procedia.

[22] Zhao X., Sun Z., Zhu Z., Li A., Li G., Wang X. (2013). Evaluation of Iron-Containing Aluminophosphate Molecular Sieve Catalysts Prepared by Different Methods for Phenol Hydroxylation. Catal. Lett..

[23] Chen J., Thomas J.M. (1994). MAPO-18 (M [Triple Bond, Length Half m-Dash] Mg, Zn, Co): A New Family of Catalysts for the Conversion of Methanol to Light Olefins. J. Chem. Soc. Chem. Commun..

[24] Mishra T., Parida K.M., Rao S.B. (1998). Transition Metal Promoted AlPO_4_ Catalyst 2. The Catalytic Activity of M_0_._05_Al_0_._95_PO_4_ for Alcohol Conversion and Cumene Cracking/Dehydrogenation Reactions. Appl. Catal. A Gen..

[25] Yadav R., Sakthivel A. (2014). Silicoaluminophosphate Molecular Sieves as Potential Catalysts for Hydroisomerization of Alkanes and Alkenes. Appl. Catal. A Gen..

[26] Wang P., Liu H., Wang C., Lv G., Wang D., Ma H., Tian Z. (2021). Direct Synthesis of Shaped MgAPO-11 Molecular Sieves and the Catalytic Performance in n-Dodecane Hydroisomerization. RSC Adv..

[27] Höchtl M., Jentys A., Vinek H. (2001). Alkane Conversion over Pd/SAPO Molecular Sieves: Influence of Acidity, Metal Concentration and Structure. Catal. Today.

[28] Deldari H. (2005). Suitable Catalysts for Hydroisomerization of Long-Chain Normal Paraffins. Appl. Catal. A Gen..

[29] Akhmedov V.M., Al-Khowaiter S.H. (2007). Recent Advances and Future Aspects in the Selective Isomerization of High n-Alkanes. Catal. Rev..

[30] Chen Z., Song W., Zhu S., Lai W., Yi X., Fang W. (2017). Synthesis of a Multi-Branched Dandelion-like SAPO-11 by an In Situ Inoculating Seed-Induced-Steam-Assisted Conversion Method (SISAC) as a Highly Effective Hydroisomerization Support. RSC Adv..

[31] Chen Z., Li X., Xu Y., Dong Y., Lai W., Fang W., Yi X. (2018). Fabrication of Nano-Sized SAPO-11 Crystals with Enhanced Dehydration of Methanol to Dimethyl Ether. Catal. Commun..

[32] Jin D., Ye G., Zheng J., Yang W., Zhu K., Coppens M.-O., Zhou X. (2017). Hierarchical Silicoaluminophosphate Catalysts with Enhanced Hydroisomerization Selectivity by Directing the Orientated Assembly of Premanufactured Building Blocks. ACS Catal..

[33] Jin D., Li L., Ye G., Ding H., Zhao X., Zhu K., Coppens M.-O., Zhou X. (2018). Manipulating the Mesostructure of Silicoaluminophosphate SAPO-11 Tumbling-Assisted, Oriented Assembly Crystallization: A Pathway to Enhance Selectivity in Hydroisomerization. Catal. Sci. Technol..

[34] Guo L., Bao X., Fan Y., Shi G., Liu H., Bai D. (2012). Impact of Cationic Surfactant Chain Length during SAPO-11 Molecular Sieve Synthesis on Structure, Acidity, and n-Octane Isomerization to Di-Methyl Hexanes. J. Catal..

[35] Fernandes A., Ribeiro F., Lourenço J., Gabelica Z. (2008). An Elegant Way to Increase Acidity in SAPOs: Use of Methylamine as Co-Template during Synthesis. Studies in Surface Science and Catalysis.

[36] Liu P., Ren J., Sun Y. (2008). Effect of Template Content on the Physicochemical Characterization and Catalytic Performance of SAPO-11 for the Hydroisomerization of n-Tetradecane. J. Fuel Chem. Technol..

[37] Luan H., Wu Q., Wu J., Meng X., Xiao F.-S. (2024). Templates for the Synthesis of Zeolites. Chin. J. Struct. Chem..

[38] Tapp N.J., Milestone N.B., Bibby D.M. (1988). Synthesis of AIPO_4_-11. Zeolites.

[39] Wang X., Zhang W., Guo S., Zhao L., Xiang H. (2013). Optimization of the Synthesis of SAPO-11 for the Methylation of Naphthalene with Methanol by Varying Templates and Template Content. J. Braz. Chem. Soc..

[40] Zhang S., Chen S.-L., Dong P., Yuan G., Xu K. (2007). Characterization and Hydroisomerization Performance of SAPO-11 Molecular Sieves Synthesized in Different Media. Appl. Catal. A Gen..

[41] Agliullin M.R., Cherepanova S.V., Fayzullina Z.R., Serebrennikov D.V., Khalilov L.M., Prosochkina T.R., Kutepov B.I. (2023). Crystallization of SAPO-11 Molecular Sieves Prepared from Silicoaluminophosphate Gels Using Boehmites with Different Properties. Gels.

[42] Agliullin M.R., Yakovenko R.E., Kolyagin Y.G., Serebrennikov D.V., Vildanov F.S., Prosochkina T.R., Kutepov B.I. (2022). Relation between Morphology and Porous Structure of SAPO-11 Molecular Sieves and Chemical and Phase Composition of Silicoaluminophosphate Gels. Gels.

[43] Serebrennikov D.V., Zabirov A.R., Saliev A.N., Yakovenko R.E., Prosochkina T.R., Fayzullina Z.R., Guskov V.Y., Kutepov B.I., Agliullin M.R. (2024). Synthesis and Application of SAPO-11 Molecular Sieves Prepared from Reaction Gels with Various Templates in the Hydroisomerization of Hexadecane. Gels.

[44] Agliullin M.R., Fayzullin A.V., Fayzullina Z.R., Kutepov B.I. (2023). The Role of Intermediate Phases in the Crystallization of Aluminophosphate Sieves on Examples of AlPO-11 and AlPO-41. Crystals.

[45] Agliullin M.R., Khairullina Z.R., Faizullin A.V., Kutepov B.I. (2019). Crystallization of AlPO4-11 Aluminophosphate from Various Aluminum Sources. Pet. Chem..

[46] Agliullin M.R., Lazarev V.V., Kutepov B.I. (2021). Influence of the Formation Conditions of Aluminophosphate Gels on the Morphology and Pore Structure of Molecular Sieve AlPO_4_-11. Russ. Chem. Bull..

[47] Agliullin M.R., Shamanaeva I.A., Zabirov A.R., Lazarev V.V., Maistrenko V.N., Kutepov B.I. (2022). Influence of the Nature of the Al Source on the Properties of the Initial Reaction Gels for Crystallization of Molecular Sieve AlPO_4_-11. Pet. Chem..

[48] Chen B., Huang Y. (2007). Examining the Self-Assembly of Microporous Material AlPO_4_-11 by Dry-Gel Conversion. J. Phys. Chem. C.

[49] Agliullin M.R., Arzumanov S.S., Gerasimov E.Y., Grigorieva N.G., Bikbaeva V.R., Serebrennikov D.V., Khalilov L.M., Kutepov B.I. (2023). Crystal Engineering of SAPO-11 Sieves by Forming Intermediate Phases. CrystEngComm.

[50] Fan F., Feng Z., Sun K., Guo M., Guo Q., Song Y., Li W., Li C. (2009). In Situ UV Raman Spectroscopic Study on the Synthesis Mechanism of AlPO-5. Angew. Chem..

[51] Holmes A.J., Kirkby S.J., Ozin G.A., Young D. (1994). Raman Spectra of the Unidimensional Aluminophosphate Molecular Sieves AlPO_4_-11, AlPO_4_-5, AlPO_4_-8, and VPI-5. J. Phys. Chem..

[52] Yu Y., Xiong G., Li C., Xiao F.-S. (2001). Characterization of Aluminosilicate Zeolites by UV Raman Spectroscopy. Microporous Mesoporous Mater..

[53] Agliullin M.R., Kutepov B.I. (2020). Selective Crystallization of AlPO4-41 Molecular Sieve in the Presence of Diethylamine. Pet. Chem..

[54] Aguado J., Escola J.M., Castro M.C. (2010). Influence of the thermal treatment upon the textural properties of sol–gel mesoporous γ-alumina synthesized with cationic surfactants. Microporous Mesoporous Mater..

[55] Araujo A., Fernandes V., Silva A., Diniz J. (1999). Evaluation of the ALPO-11 Crystallinity by Thermogravimetry. J. Therm. Anal. Calorim..

[56] Wang W., Liu C.-J., Wu W. (2019). Bifunctional Catalysts for the Hydroisomerization of N-Alkanes: The Effects of Metal–Acid Balance and Textural Structure. Catal. Sci. Technol..

